# Changing techniques in cataract surgery: how have patients benefited?

**Published:** 2018-02-08

**Authors:** Aravind Haripriya, Hemant Sonawane, RD Thulasiraj

**Affiliations:** 1 Chief: Cataract services, Aravind Eye Care System, Chennai, India.; 2 Medical Consultant: Aravind Eye Care System, Madurai, India.; 3 Executive Director: Aravind Eye Care System, Madural, India.

**Cataract surgery is one of the most frequently performed operations in the world. Recent advances in techniques and instrumentation have resulted in earlier intervention, improved surgical outcomes, and reduced dependence on spectacles.** ^1^

**Figure:**
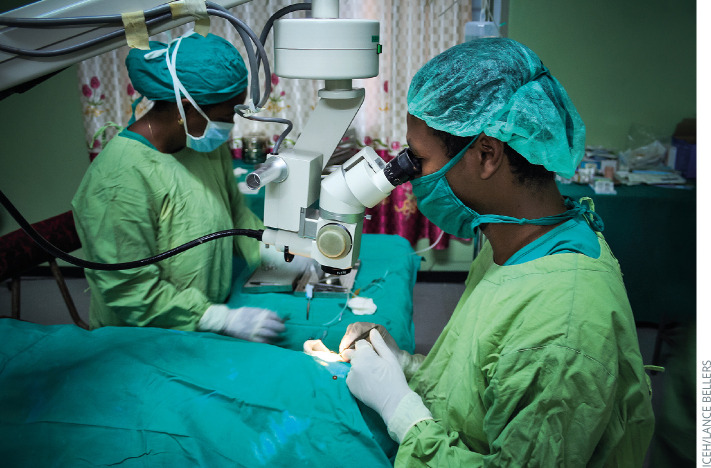
Cataract surgery is essential in order to address avoidable blindness. ETHIOPIA

The first record of cataract being surgically treated is by Susruta, who carried out the procedure in 600 BC. Cataracts were treated using a technique known as couching, in which the opaque lens is pushed into the vitreous cavity to remove it from the visual axis. Couching is still performed in some parts of Africa and the Middle East. In 1753, Samuel Sharp performed the first **intracapsular cataract extraction** (ICCE) through a limbal incision. He used pressure from his thumb to extract the lens. In 1961, Polish surgeon Tadeusz Krwawicz developed a cryoprobe which could be used to grasp and extract cataracts during ICCE surgery. However, an aphakic spectacle correction was still required. When the first edition of the *Community Eye Health Journal was* published, ICCE was still the most widely practised method of cataract extraction in low- and middle-income countries. However, in high-income countries, ICCE had been superseded by extracapsular surgery with an IOL implant.

Modern **extracapsular cataract extraction** (ECCE) gained acceptance in high-income countries after the introduction of operating microscopes during the 1970s and 1980s made it possible to perform microsurgery. The microscopes offered better intraocular visibility and the ability to safely place multiple corneal sutures. ECCE has the advantage of leaving the posterior capsule intact; this reduces the risk of potentially blinding complications and makes it possible to implant a lens in the posterior chamber.

**Phacoemulsification** was introduced in 1967 by Dr Charles Kelman. Since then, there have been significant improvements in the fluidics, energy delivery, efficiency and safety of this procedure. Advantages include small incision size, faster recovery and a reduced risk of complications.^2^

**Manual small-incision cataract surgery** (MSICS) is a small-incision form of ECCE with a self-sealing wound which is mainly used in low-resource settings. MSICS has several advantages over phacoemulsification, including shorter operative time, less need for technology and a lower cost.^1^,^2^ It is also very effective in dealing with advanced and hard cataracts. As with modern ECCE techniques, MSICS also allows for a lens to be implanted.

A recent introduction is **femtosecond laser-assisted cataract surgery,** during which a laser is used to dissect tissue at a microscopic level. Initial results from the recent FEMCAT trial suggest little or no improvement in safety and accuracy compared to standard phacoemulsification, and the procedure brings with it new clinical and financial challenges.

Today, although phacoemulsification is considered the gold standard for cataract removal in high-income countries, MSICS is hugely popular and practised widely in many countries of the world because of its universal applicability, efficiency and low cost.^3^

## Improvements in ophthalmic equipment and intraocular lenses

Over the three decades since the first issue of the *Community Eye Health Journal* was published, the availability of microsurgery and high-quality intraocular lenses (IOLs), at an acceptable cost, have made a positive global impact on visual results after cataract surgery.

IOLs can be placed in the anterior chamber or posterior chamber, or be supported by the iris. The preferred location is the posterior chamber, where the posterior chamber IOL (or PCIOL) is supported by the residual lens capsule.

Sir Harold Ridley is credited with the first intraocular lens implantation in 1949, using a material known as PMMA. Since then, numerous design and material modifications have been developed to make IOLs safer and more effective, and they have been in routine use in high-income countries since the 1980s. However, when the first edition of the CEHJ was published in 1988, an IOL cost approximately $200 and was far too expensive for widespread use in low- and middle-income countries. Thankfully, owing to the foresight and innovation of organisations such as the Fred Hollows Foundation and Aravind Eye Hospitals, IOLs are now produced at low cost in low- and middle-income countries and have become available to even the most disadvantaged patients.

**Table 1:** Guidelines for the choice of formula based on axial length and specific circumstances

Circumstance		Recommended fomulae
Axial length	<22mm	Haigis/Hoffer-Q
	22–26mm	SRK-T
	>26mm	Haigis/SRK-T
Myopic LASIK		Haigis-L, ASCRS online calculator
Following radial keratotomy		ASCRS online calculator
Use of a piggyback IOL		Holladay's refractive formula
Adapted from **www.nice.org.uk/guidance/ng77/chapter/Recommendations#preoperative-assessment-and-biometry** (Oct 2017)		

With the introduction of the first **multifocal and toric IOLs,** the focus of IOL development has shifted toward improving refractive outcomes and reducing spectacle dependence. Toric lenses correct postoperative astigmatism, and multifocal lenses reduce dependency on spectacles for near vision. However, multifocal lenses may cause glare and reduced contrast sensitivity after surgery and should only be used in carefully selected patients. The **accommodating lenses** that are in current use are limited by their low and varied amplitude of accommodation.

The **light-adjustable lens** is made of a photosensitive silicone material. Within two weeks of surgery, the residual refractive error (sphero-cylindrical errors as well as presbyopia) can be corrected by shining an ultraviolet light on the IOL through a dilated pupil to change the shape of the lens. Development of an **intraocular lens (IOL) as a drug delivery device** has been pursued for many years. Common postoperative conditions such as posterior capsular opacification (PCO), intraocular inflammation or endophthalmitis are potential therapeutic targets for a drug-eluting IOL.

## Choosing the best IOL

Although inserting a standard power IOL in all eyes is an improvement over standard power aphakic spectacles, the best results are obtained by calculating the correct power of lens implant for each eye. Many formulae have been developed over the last three decades to calculate the IOL power; however, no single formula works well in all circumstances. Table 1 provides guidelines for the choice of formula based on axial length and specific circumstances.

## Changing trends in anaesthesia techniques

Ocular anaesthesia has evolved tremendously since Einhorn synthesised procaine in 1905, which led to its acceptance in retrobulbar anaesthesia. Drs David and Mandal introduced peribulbar anaesthesia in 1980. However, in the last two decades, sub-Tenon's anaesthesia has become a common technique for ocular anaesthesia. By using a blunt cannula to deliver the local anaesthetic, it avoids the risk of accidental perforation of the globe, which is a serious complication of retrobulbar and peribulbar anaesthetic techniques. There is a trend towards non-needle local anaesthesia techniques^4^, which have the advantage of avoiding completely the complications related to orbital injections. The drawbacks include reliance on patient cooperation.

## Adjuncts in cataract surgery

Over the last two to three decades, numerous adjuncts have been developed to improve outcomes in routine and complex cataract surgery. Use of dispersive and cohesive **ophthalmic visco-surgical devices** (OVDs) have led to a dramatic improvement in the safety of cataract surgery.^5^ In addition, capsular tension rings, iris retractors and pupil expansion devices have made cataract surgery safer in eyes compromised by weak zonules, small pupils or a floppy iris.

## A public health triumph

Clinical advances have led to significant improvements in cataract outcomes. Similarly, low-cost IOLs, surgical skill development and community-oriented programmes, together with government funding and enabling policies, have also dramatically increased the number of people who are able to benefit from cataract surgery.

## Future outlook

The future of cataract treatment promises to be exciting. Surgery may not be the only treatment option if research can identify an agent to either slow or reverse lens opacification. For the immediate future, however, lens surgery will remain relevant for the world's ageing population.

**Figure:**
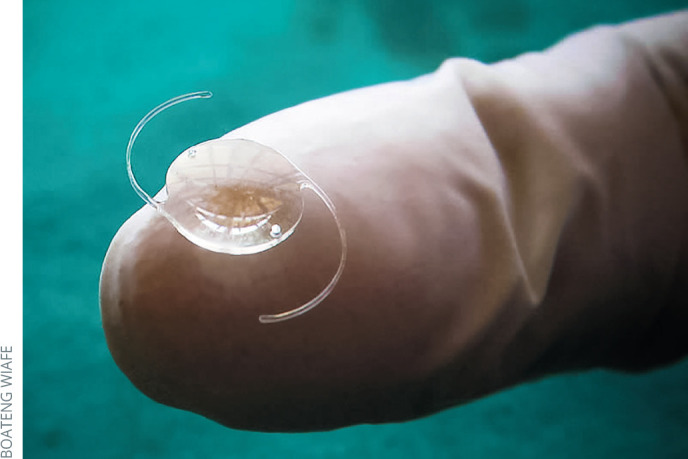
The arrival of affordable IOLs has radically improved visual outcome after cataract surgery. GHANA

## References

[1] Alkharashi M Stark WJ Daoud YJ. Advances in cataract surgery. Expert Rev Ophthalmol 2013;8(5):447–56.

[2] Kongsap P. Visual outcome of manual small-incision cataract surgery: comparison of modified Blumenthal and Ruit techniques. Int J Ophthalmol 2011;4(1):62. 22553611 10.3980/j.issn.2222-3959.2011.01.14 PMC3340689

[3] Venkatesh R Chang DF Muralikrishnan R Hemal K Gogate P Sengupta S. Manual Small Incision Cataract Surgery: A Review. Asia Pac J Ophthalmol (Phila) 2012;1(2):113–9. 26107133 10.1097/APO.0b013e318249f7b9

[4] Lee RM Thompson JR Eke T. Severe adverse events associated with local anaesthesia in cataract surgery: 1-year national survey of practice and complications in the UK. Br J Ophthalmol 2016;100(6):772–6. 26405103 10.1136/bjophthalmol-2015-307060

[5] Haripriya A Chang DF Namburar S Smita A Ravindran RD. Efficacy of intracameral moxifloxacin endophthalmitis prophylaxis at Aravind Eye Hospital. Ophthalmology 2016;123(2):302–8. 26522705 10.1016/j.ophtha.2015.09.037

